# ‘A Forever Imprint on Me…I Couldn't Be There When He Needed Me the Most’: Care Partner Experiences of Patient Safety During Covid‐19 Restrictions

**DOI:** 10.1111/hex.70496

**Published:** 2025-11-18

**Authors:** Kayley Perfetto, Kim Sears, Jane O'Hara, Lenora Duhn

**Affiliations:** ^1^ Health Quality Programs, School of Nursing Queen's University Kingston Ontario Canada; ^2^ School of Nursing Queen's University Kingston Ontario Canada; ^3^ THIS Institute Cambridge University Cambridge UK

**Keywords:** care partners, Covid‐19 pandemic, engagement, patient safety, qualitative study, visitor restrictions

## Abstract

**Background:**

It is believed that engaging care partners (CPs) in patient safety is a potential strategy to ensure patients' well‐being during hospital admissions. When present, CPs who feel comfortable can be ‘safety nets’, monitoring and acting to prevent healthcare‐related harm. However, there are limitations in evidence about CPs' perspectives regarding and experiences of safety engagement at the direct care level and the implications if they have restricted access, such as during the Covid‐19 pandemic.

**Objective:**

To understand CPs' experiences of patient safety when, due to Covid‐19 restrictions, they were unable to be present in the hospital during a patient admission.

**Methods:**

A descriptive, exploratory qualitative study was conducted in an acute care hospital in Ontario, Canada, and four Canadian organisations that support CPs. One‐to‐one semi‐structured interviews and reflexive thematic analysis were completed.

**Findings:**

Twelve CPs participated. Overall, they believed hospital restrictions had consequences on patient safety and was troubling for them and their loved ones, as illuminated in the three main themes: *Stress Escalates Amid Disrupted, Ineffective Communications Between CPs and Patients/Healthcare Professionals; CPs' Patient Safety Concerns Change and are Heightened in Their Absence From the Bedside, Calling for New Tactics*, and *Hospital Access Restriction Negatively Impacts CPs' Well‐Being, Necessitating Creative Ways to Bolster Their Strength*.

**Discussion:**

The findings give insight into CPs' engagement in patient safety at the direct care level and the importance of being present, given the types of contributions they want to make. Communication was paramount, and the scope of their involvement—the nuances of which became more apparent in their absence—affirmed how connected CPs are and need to be within the health team for successful risk reduction. Disconnecting CPs from engaging in safety diminished the patient's protection, and instead of reducing CP fears related to involvement, it escalated newfound worries and stressors.

**Conclusion:**

This study highlights CPs' experiences with patient safety when they cannot be at the bedside and their crucial role in mitigating risks, and, in anticipation of future public health crises, as well as ongoing care approaches, questions the risk in prohibiting and not fostering CPs' involvement at the bedside.

**Patient or Public Contribution:**

Twelve CPs of adult patients with lived experiences of engaging in patient safety participated in this study. These CPs were unable to be present with the patient during their admission due to visitor restrictions implemented as a result of Covid‐19.

## 1 Introduction

Care partner (CP) is defined as any unpaid, patient‐identified person(s) who a patient wishes to be involved in their care and act on their behalf and may include unrelated individuals [1]. *CP engagement in patient safety* is endorsed as an essential strategy for improving safety in healthcare [2, 3]. When present for an adult patient's hospital admission, CPs can foster safety by monitoring for healthcare harm and acting to protect the patient [4, 5, 6]. It is believed a CP's ability to engage in safety‐related behaviours is dependent, in part, on their presence at the patient's bedside—for example, certain types of monitoring would not be possible without being present [4]. During the Covid‐19 pandemic, the decision to change normal practice and restrict CPs from hospitals hindered their ability to participate in safety activities, potentially increasing the risk of patient harm [7]. As health systems learn from the Covid‐19 pandemic, decisions regarding CP's presence in hospitals must not have unintended consequences on patient safety. As such, it is important to understand the experiences of CPs when they were prohibited from being at the bedside, including insights into the implications of CPs' physical absence and patient safety; such evidence will contribute to improved care‐partner‐presence policies and patient safety at the direct care level.

## 2 Background

Patient safety involves minimising and preventing harm or adverse events (AEs) in healthcare, as well as learning from such events when they occur [2]. Canadian researchers estimate that nearly one in four Canadian hospital admissions results in an AE each year [8]. An AE is an incident that causes unintended patient harm resulting from health services; it is not a known treatment complication, nor does it result from the patient's medical condition [1]. AEs can have a profound impact on patients and their families, leading to unexpected medical interventions, increased length of stay and/or physical limitations [9, 10]. Following an AE, patients and their families may feel angry or helpless, potentially leading to a reluctance to seek future medical care [9].

CPs can help prevent AEs. During their hospital admission, a patient may feel anxious or unable to advocate for safe care [11, 12]. Further, they may prefer to be passive recipients of care [13, 14]. In these instances, patients may delegate this responsibility to a CP, having them advocate on their behalf [13, 14, 15]. CPs report feeling responsible for accepting this role, monitoring the care and raising concerns [16]. CP presence is particularly important for groups at increased risk of an AE, including elderly patients or those with impaired cognitive function [16, 17].

Patients report feeling safer when CPs are present [12, 18], with some experiencing anxiety in their absence, fearing they are not receiving safe care [18]. CPs can be well‐informed patient advocates due to their knowledge of the patient's condition [12] and can facilitate communication with the healthcare professionals (HCPs), clarifying information and preventing medication misunderstandings [16, 18, 19, 20]. While this information has been informative, much of the research has not included CP perspectives independent of the patient, nor has it been within the context of the CP at the direct care level. A better understanding of CP experiences in patient safety is needed to effectively develop and implement engagement programmes. While there is evidence that CPs feel their role is to be present at the patient's bedside, their beliefs and preferences about this involvement remain inadequately understood. Additionally, the actions CPs undertake to ensure the safety of patients are not clearly defined. This information is important if evidence‐informed policies and safety initiatives are to reflect the voice and preferences of CPs.

The Covid‐19 pandemic presented a unique opportunity to understand the implications of CP's absence at the direct care level on patient safety. In March 2020, the World Health Organization (WHO) declared the Covid‐19 virus a global pandemic [21, 22]. To ensure the safety of patients and healthcare workers, hospitals implemented emergency solutions, including restricting CPs' access [21, 23]. Restricting CPs from the patient's bedside created a ‘natural experiment’ to explore the impact of CP absence on patient safety. There has been some exploration of the experiences of CPs during the pandemic, for example, the increased use of telephone calls and virtual ‘visits’ to communicate with patients and HCPs, creating inequities for families without electronic devices or limited digital literacy [21, 24]. Having to depend on telephone and video calls to communicate with HCPs caused frustration for some CPs, as they found it difficult to understand the information [25], raising their doubts about the quality of the care [26]. The absence of CPs presented a challenge when the patient could not verbally communicate, leading to potentially ineffective communication between the patient and the HCP [25, 27, 28]. Further, evidence suggests that CPs experienced stress due to their inability to advocate for the patient during their illness [7, 29].

The necessities of managing the ‘unknowns’ of Covid‐19 presented an unprecedented opportunity to examine the role of CP in supporting safety, through its absence, that would not have been possible, and certainly unethical, in ordinary circumstances. Given the limited empirical evidence for CPs' perspectives on patient safety and their involvement in it, the present study aimed to explore: *What do CPs of adult patients reveal about their lived experiences of safety on inpatient hospital units with restricted CPs' presence/visiting hours due to Covid‐19, and their beliefs about their involvement in patient safety at the direct care level?*

## 3 Methods

### 3.1 Study Design

This study is part of a multiphase study, which includes a qualitative systematic review about experiences of CP engagement in patient safety at the direct care level from CP, patient and HCP perspectives [placeholder]. It is approached from the constructivist paradigm, which posits that multiple socially constructed realities exist, shaped by an individual's previous experiences as opposed to one universal truth [30, 31], and underpinned by two theoretical perspectives, *A Multidimensional Framework for Patient and Family Engagement in Health and Healthcare* [32] and the *5‐Facet Framework for Patient Engagement in Patient Safety* [19]. These frameworks informed the entirety of the study by defining relevant concepts, developing the research question and guiding the study design and were used during data analysis, guiding the development of relevant themes [33]. The qualitative design is exploratory descriptive, which is appropriate given the goal of developing an in‐depth understanding of a phenomenon from the perspective of those who experienced it [34].

### 3.2 Study Participants and Setting

CPs were defined as anyone the patient wanted involved in their medical care and acted on their behalf, was unpaid for this support, and may or may not be related to the patient [1]. To be eligible, participants had to be: (1) a CP of an adult patient (18 years of age or older); (2) a CP of a patient admitted to an inpatient unit with CPs' presence restrictions (due to Covid‐19) for at least one night; (3) 18 years of age or older; (4) able to provide consent; and (5) able to speak, read and understand English. CPs were excluded if: (1) the patient, the CP was caring for, was admitted to palliative care, surgical day care or an obstetrics unit, as these were essential visitors and exempt from restrictions [35] or (2) the patient was younger than 18 years of age, as this is not the study focus. Participants were recruited through purposive sampling in two ways. First, CPs were recruited from the Patient and CP programme, a group of volunteer community advisors, at a 1000‐bed, acute care hospital in Ontario, Canada. Second, CPs were recruited through four not‐for‐profit, provincial and national organisations that support CPs [36, 37, 38, 39]. Study information was shared with group members, and those interested were invited to contact the primary researcher, who screened them for eligibility.

### 3.3 Interview Tool

Semi‐structured interviews were conducted to gain an in‐depth understanding of CPs' experience when they were unable to be with the patient during their hospitalisation. The questions were informed by the two aforementioned theories and professional knowledge to explore the research question. Table 1 presents the interview questions. The questions were written at the Flesch Kincaid grade level 5 [40]. Demographic questions were asked to describe participant characteristics.

**Table 1:** Interview questions.

**Demographic questions**
1. Can you please tell me your age in years?
2. Can you please tell me about your relationship with the patient?
3. Can you please describe your gender identity?
4. Can you please describe your ethnicity?
5. What is the highest level of education completed?
**Interview questions**
1. To start, can you tell me a little bit about yourself?
2. How would you describe your role as a care partner?
3. What has the experience of safety, specifically, keeping patients safe while they are admitted to the hospital, been like when you were not able to be at the patient's bedside? An example of safety is asking a care provider if they have washed their hands before seeing your loved one.
4. How do you describe the safety of the patient during their admission?
5. Were there any activities or things that you did to promote the safety of the patient while they were in the hospital?
6. Have you ever experienced healthcare errors, either during this admission or a previous admission?
7. Can you describe your well‐being during this time (when unable to be with the patient during their hospital admission due to visitor restrictions)?
8. How do you describe your role in patient safety for your loved one at the direct care level?
9. Is there anything else that you would like to tell me about your experiences?

### 3.4 Procedure

The associated university research ethics board granted ethics approval for this study (HSREB [placeholder]). Following the eligibility screening, the study was described to the participant, questions were answered, consent was obtained, and interviews were scheduled with interested participants. Interviews were conducted in person (such as at the participant's home), virtually or by telephone, based on participant preference, in the winter of 2024. All interviews were video‐ and/or audio‐recorded. To allow for reflection on the interview questions and preparation of responses, participants were emailed the questions in advance, along with a list of support organisations should the interview evoke an emotional response. All interviews lasted between 45 and 60 min. Participants were given the option of a second interview to discuss the interview transcript, which was emailed to them for review. One of the 12 participants participated in a second interview, which led to clarifying edits in the transcript. Participants were provided with a pseudonym to maintain their privacy.

### 3.5 Data Analysis

Each interview was transcribed verbatim, using Otter transcription software [41] and proper nouns were removed. Then, the interview transcripts were analysed using reflexive thematic analysis (RTA) [33]. RTA is a six‐step process, beginning with the familiarisation of the dataset and involving numerous reviews of the transcripts [33]. Then, codes were generated and data sections relevant to the research question were marked with a code label [33]. Similar codes were grouped to establish an initial set of themes [33]. Themes were refined to ensure they were cohesive and addressed the research question [33]. Themes were then defined and named [33].

### 3.6 Trustworthiness

The Model of Trustworthiness was used to uphold the quality, rigour and integrity of the research, with careful consideration given to the criteria of credibility, transferability, dependability and confirmability [42]. Table 2 presents the details about trustworthiness strategies.

**Table 2:** Model of trustworthiness.

Concept	Actions
Credibility	Adequate time was spent with each participant to develop rapport, fostering trust and encouraging open sharing [5, 43, 44] Participants were provided the option of a second interview to review the transcript and confirm its accuracy [5, 43, 44] The primary researcher completed reflexive journaling following each participant interview [5, 43] Regular discussion with the research team regarding the study process [5, 43, 44] Member checking: participants were asked to review transcripts for accuracy [5, 44] The primary research engaged in peer examination by discussing the research process and findings with research colleagues [5] Participant interviews followed a researcher guide, which allowed for consistency between interviews [5, 43, 44]
Transferability	Demographic questions were asked to provide a detailed and dense description of the sample [5, 43] Verbatim quotes from participants were utilised to allow for a clear understanding of the participants' experiences [43]
Dependability	The study process was documented in great detail [5, 43, 44] The accuracy of transcripts was confirmed with participants [5, 43, 44]
Confirmability	There was detailed note‐taking of decisions made, creating an audit trail [5, 43, 44]

## 4 Findings

Ten women and two men were in this study, and their ages ranged from 39 to 89 years (average age = 60 years; median age = 70 years). One participant was screened and did not meet the inclusion criteria and was not included. Our analysis indicated that after the 12 participant interviews, no new codes were identified, information power was achieved [45], and recruitment was concluded [46]. All individuals could not visit patients admitted to the hospital due to Covid‐19 restrictions, except for three participants who had limited access after the patient was palliative. Nine individuals described when their spouse (eight husbands and one wife) was admitted, and three detailed a parent's admission (two mothers and one father). Three patients were transported from their homes to the hospital via ambulance after the CP called 911. Patients' length of stay ranged from 5 days to 6 weeks. Three patients died while in the hospital. Seven patients had pre‐existing medical conditions; these CPs related to pre‐ and Covid care experiences. Five patients did not have pre‐existing medical conditions, and the participant did not support the patient's medical needs before this admission. Three CPs identified as being HCPs and were working or had worked in a hospital; two of these individuals assisted patients with a pre‐existing medical condition. Table 3 displays patient characteristics.

**Table 3:** Characteristics of care partners (CPs) and patients.

Pseudonym	Gender F = Female M = Male	Age (years)	Level of education	CP's relationship to the patient	Patient's reason for admission	CP reports patient had an AE during admission (Yes/No)	CP is/was an HCP (Yes/No)	The patient has a chronic condition (Yes/No)
Audrey	F	70	Graduate Degree	Wife	Stroke	No	No	Yes
Bob	M	89	Graduate Degree	Husband	Cancer	No	Yes	Yes
Rose	F	85	High School	Wife	Stroke	No	No	Yes
Brenda	F	72	University Degree	Wife	Respiratory	No	No	No
Nancy	F	75	University Degree	Wife	Exacerbation of Parkinson's disease	Yes	No	Yes
Jennifer	F	58	College Diploma	Daughter	Hip fracture	Yes	No	No
Dianne	F	70	University Degree	Wife	Exacerbation of Alzheimer's disease	No	Yes	Yes
Amanda	F	39	Graduate Degree	Wife	Brain bleed	No	Yes	No
Shane	M	40	College Diploma	Son	Cancer	No	No	No
Teresa	F	70	University Degree	Wife	Pneumonia	Yes	No	Yes
Rebecca	F	50	Graduate Degree	Wife	Cancer	No	No	No
Andrea	F	61	University Degree	Daughter	Dementia	Yes	No	Yes

*Note:* All participants identified as White.

Abbreviations: AE—adverse event; CP—care partner; HCP—healthcare professional.

Three overarching themes, with related sub‐themes, were created:

- **Theme 1**: Stress Escalates Amid Disrupted, Ineffective Communications Between CPs and Patients/HCPs [sub‐themes: *CPs' Stress From Limited Communication With Patients; CPs Speaking With HCPs—The Importance, Challenges and Workarounds*];
- **Theme 2**: CPs' Patient Safety Concerns Change and are Heightened in Their Absence From the Bedside, Calling for New Tactics [sub‐themes: *Additional and New Patient Safety Worries for CPs in an Unfamiliar, Restricted Context; Exposure to Adverse Events: The Loss of the CP's Protection; ‘I Insisted…. Just to Make Sure’: CPs' Resourceful Strategising to Reduce Harm*]; and
- **Theme 3**: Hospital Access Restriction Negatively Impacts CPs' Well‐Being, Necessitating Creative Ways to Bolster Their Strength [sub‐themes: ‘*A Forever Imprint on Me—I Have Never Felt So Useless’: Deep and Lasting CP Emotions and Physical Toll; ‘I Needed to Stay Close, In Case’: Resilience of CPs and Their Coping Strategies]*.

Table 4 displays supplementary participant quotes regarding themes/sub‐themes.

**Table 4:** Supplementary participant quotes.

Theme 1: Stress Escalates Amid Disrupted, Ineffective Communication Between CPs and Patients/HCPs	
Theme/Sub‐theme	Participants' verbatim words
CPs' Stress From Limited Communication With Patients	*When I couldn′t get a hold of him, I was, like, he′s had a complication, he not conscious … he can′t answer his phone because he′s not doing well … if I couldn′t get a hold of him I was convinced he was incapacitated and something had happened* (Amanda) *And then I would call and get no answer, and then I would panic. I would panic because he wasn′t answering his phone and of course, my anxiety was already ratcheted on a scale of 1 to 10 about a 15…. I would go into full panic mode, there′s been a setback, he′s gone septic*. (Rebecca)
CPs Speaking with HCPs—The Importance, Challenges and Workarounds	*I phoned a minimum of twice a day and every time I talked to a different nurse who didn′t seem to have any history of her. They were short‐staffed so I had to mention my concerns every time I spoke to someone—like, is she taking her meds?* (Andrea) *I have to be very honest, I never, the entire time he was there, spoke to like a doctor or anything. I also felt guilt about bothering the nurses … well, if there′s something wrong with him the nurses are going to be busy* (Amanda)
‘By Not Being There I Had No Idea What Was Going On’: CPs Feel the Need and Want the Chance for Communication	*It was just hard to uh keep up with what was happening* (Bob) *Like my husband doesn′t ask many questions, but if I had been there, I would have been like for sure … well what can he do? What should we look out for? When should we come back?* (Amanda)

### 4.1 Theme 1: Stress Escalates Amid Disrupted, Ineffective Communications Between CPs and Patients/HCPs

Theme 1 is about the disruptions to communication and exchange of information that CPs experienced because they were not present with the patient. The consequences of this change meant CPs felt they could not effectively communicate with the patient and/or the HCP. Being separated from the patient during their admission hindered the CPs' ability to see for themselves, which was essential to understand the patient's medical condition and confirm their safety and well‐being. Communication with the HCPs and the patient served as a crucial link for the CP, and when limited, it raised concerns regarding the patient's recovery and the delivery of safe care, resulting in increased stress for the CP.

#### 4.1.1 CPs' Stress From Limited Communication With Patients

CPs shared difficulties and frustrations in communicating with admitted patients. Some patients were unable to speak after being placed on a ventilator or because of new medications. Bob shared how a previous stroke affected his wife's ability to speak on the phone, emphasising the importance of in‐person communication. Several CPs reported that despite insisting the patient take a personal communication device (i.e., cell phones) with them, they could not use them due to difficulties operating the technology, or their illness made them too unwell to speak. Amanda shared how ‘facetiming’ with her husband was not possible, as the screen time bothered his head injury. Another patient's diagnosis of Parkinson's disease made it impossible to hold a phone, while another patient simply did not use technology. Some patients required assistance to charge their phones or call the CP, and due to the significant work demands of HCPs, they often could not help. Additionally, patients with dementia found the use of electronics confusing, as they did not understand why their CP could not be there in person. This inability to communicate with the patient—who often did not understand the reasons behind it—created significant worry for the CP, making them fear the worst about their loved one. Amanda revealed that not being able to speak with her husband caused her

*to spiral, like something had happened to him*,

as she assumed he was not answering his phone because of a medical complication. CPs shared that in the absence of being physically present with the patient, the ability to regularly speak with them would have provided reassurance about their condition and eased their safety concerns.

#### 4.1.2 CPs Speaking With HCPs—The Importance, Challenges and Workarounds

CPs had difficulty communicating with HCPs, expressing frustration over having to rely on telephone calls for patient information, missing the in‐the‐moment, face‐to‐face communication that occurred when they were physically present. CPs indicated many HCPs did not contact them during the patient's admission, leaving CPs feeling disconnected from the patient and their care. Teresa said that, although she requested a telephone call when her husband was out of surgery, she did not receive one, and it was her husband who called her with an update from the Post‐Anaesthesia Care Unit. Several CPs remarked that accessing information would not have been an issue had they been present, as exemplified by Nancy, who stated:

Even though we were having a [virtual] family conference once a week … face‐to‐face makes a difference when you're asking questions about the patient and you're getting information … you really lost that general flow, if I were visiting him every day I would have been talking to the nurses.

Additionally, CPs hesitated to contact HCPs for updates, for fear of diverting attention from patient care. Some CPs, including those who are HCPs, believed the pandemic increased demands on HCPs, leaving them with little time to communicate with families. Overall, these communication barriers resulted in uncertainty, raising concerns about the quality of care; it seems the ‘unknown’—no ability to see or talk with a patient or an HCP—is a significant stressor and derails CPs' ability to make a determination about a patient's circumstance. This finding highlights the significance of the connection between HCPs and CPs, which enables CPs to continue to advocate for the patient's safety. Notably, some CPs described communication enablers for speaking with HCPs, such as scheduling specific times to call. Nancy spoke with the HCP every evening at 10:00 PM to receive patient updates; this prevented repeated calls.

#### 4.1.3 ‘**By Not Being There I Had No Idea What Was Going On’: CPs Feel the Need and Want the Chance for Communication**

As a consequence of inconsistent communication with HCPs and patients, CPs found it difficult to understand the patient's medical condition and the treatment provided. CPs described how the inability to connect with the patient or their HCP left them with unanswered questions, uncertainties about the patient's diagnosis, and worries about the patient's recovery. Amanda described,

And just not even having any idea…. How is he doing? Is this what is expected? Or being able to ask more questions about … well, you found nothing? Like is that good? Is that bad? … is he at risk for it later?… By not being there, I would say that I had no idea what was going on.

One CP shared she did not know her husband had a stroke until he was discharged home, while another CP was not aware the patient, due to increased care needs, was moved to a different inpatient unit. Bob recalled the difficulties he faced in attempting to discuss his wife's condition with her oncologist, and despite his multiple attempts, he was unable to do so during her admission. CPs felt that without a thorough understanding of their loved one's condition and treatment, they could not effectively advocate on their behalf, increasing their risk of healthcare harm.

### 4.2 Theme 2: CPs' Patient Safety Concerns Change and Are Heightened in Their Absence From the Bedside, Calling for New Tactics

This theme is about the increased and new safety concerns CPs had as a result of not being with the patient during their hospital admission. Many CPs noted that being separated during the patient's admission raised their ‘normal’ concerns about patient safety, which may not have arisen had they been present. It also included new issues, specifically, something that would not be relevant if they were present, but became problematic in their absence (e.g., is the patient taking their medications). By not being present with the patient, CPs missed the opportunity to monitor the quality of care and the patient's condition, raising significant concerns about the safety of care.

One important implication of CPs being away from the bedside was the occurrence of AEs, which CPs believed could have been prevented if they had been there. Despite being physically separated, CPs took measures to augment patient safety during the patient's admission.

#### 4.2.1 Additional and New Patient Safety Worries for CPs in an Unfamiliar, Restricted Context

CPs shared that not being present with the patient during their admission led to increased concerns for patient safety. By restricting CPs from the patient's bedside, they were no longer positioned to monitor the quality of care, the risk of harm, or changes in the patient's condition. Teresa was afraid her husband was not wearing his dentures and would choke on food that was improperly cut. Andrea worried her loved one, who lives with dementia, would wander away from their room and become injured. Several CPs, whose loved ones were challenged with communication, were anxious that those individuals would be unable to voice concerns about their care to their HCPs or advocate for themselves. Audrey, whose loved one had a tracheostomy tube, worried the HCPs would not know how to care for it, revealing:

…most, nurses and some doctors, are not familiar with the condition he is dealing with. And that was the case when he went in with his stroke, he was getting blocked up, but they didn't know that was what was happening.

Audrey felt that had she been there, she would have provided the HCPs with the needed information. CPs also worried that patients, due to illness or otherwise, were not questioning the care provided by the HCPs, trusting that it was safe. Additionally, in light of the restrictions, patients underwent medical interventions and/or received distressing news (e.g., cancer diagnosis) without CP support, leading to concerns about their emotional well‐being. Amanda's safety concerns for her husband increased after he received an upsetting diagnosis, which she felt impacted his state of mind. All these concerns were compounded by fears of the patient contracting Covid‐19. Several patients informed their CPs they felt overwhelmed and vulnerable by managing their care alone and unable to protect themselves—which further exacerbated CPs' worries and affirmed their belief in the need to be engaged, however that might occur.

#### 4.2.2 Exposure to AEs: The Loss of the CP's Protection

CPs recounted how the patient experienced an AE during their hospital admission—events the CPs felt they could have prevented had they been present. Nancy described her terrifying experience when her admitted husband repeated, *No, no, no*, during a telephone conversation. Fearing what was occurring, but knowing she needed to help, Nancy recounted what she said and did:

[imparting what she said to her husband] ‘I don't know what's going on, but I'm gonna deal with it. You need help. Something's going on … don't worry, I'm going to have you hang up, and I'm going to call the nurse's station and see if somebody needs to be there for you.’ I called the nurse's station, and I said, I don't know what's going on with my husband, but something's really going on.

The HCP later informed Nancy her husband had been attempting to pull out his feeding tube, something Nancy felt could have been prevented had she been there. Several CPs echoed Nancy's thought that AEs could have been prevented had they been present. One patient experienced aspiration pneumonia after eating while lying down; another patient with dementia developed a bladder infection due to inadequate hydration; and another patient suffered repeated falls from their bed. Jennifer recalled how she had received a call from her father's physician explaining how her father had crawled over the bedrails, fallen to the floor and fractured the hip that had just been surgically repaired.

#### 4.2.3 ‘I Insisted…. Just to Make Sure’: CPs' Resourceful Strategising to Reduce Harm

Despite being physically distanced, CPs were not deterred by their absence from the patient and employed strategies to keep the patient safe. After realising the HCPs were not providing regular patient updates, many CPs took the initiative to facilitate communication with them. Audrey described:

So I insisted that every time a nurse or a doctor came into his room he would say, just one moment, I need to call my wife and put you on speaker so I could hear every communication they were having.

Bob resorted to writing a letter and requested a written response from the physician about his wife's condition. Several CPs, concerned that an increased workload due to the pandemic may cause poor communication between HCPs, contacted the patient's family physician to inform them of the patient's hospital admission. This also eased patients' concerns about their treatment, and in some cases, led to hospital visits and treatment from their other HCPs (e.g., endocrinologist). To understand the patient's diagnosis and appropriately advocate on their behalf, some CPs requested the HCPs read the patient's chart over the telephone, while others leveraged the expertise of HCPs in their family, having them provide questions for the HCP and help them to understand the patient's conditions. Also, as able, CPs provided patients with questions to ask HCPs—an approach that reduced the burden of CPs repeatedly contacting HCPs and facilitated the collection of valuable patient information. When they were able to speak with the patient, CPs provided advice, including encouraging them to take their medications. Brenda advised her husband, who was admitted with a respiratory condition, to avoid talking due to his difficulty breathing, even though it meant sacrificing their daily phone calls. To prevent contracting Covid‐19, Rebecca instructed her husband to always wear a mask. Jennifer advocated to the HCPs to have restraints used with her father; she recalls pleading with the HCP,

Please tie him in the bed, you have the family's approval, he is known to wander, it is for his own safety.

Other CPs advocated and taught HCPs about the unique patient care needs to avert safety issues and reduce risk, which was particularly important for patients with reduced cognitive function.

### 4.3 Theme 3: Hospital Access Restriction Negatively Impacts CPs' Well‐Being, Necessitating Creative Ways to Bolster Their Strength

This theme is about how restricting CP in‐hospital presence due to Covid‐19 and the need to adjust to new circumstances impacted the well‐being of CPs, as well as coping strategies. CPs felt the need to be with their loved ones during their hospitalisation, and the inability to do so had a significant emotional and physical impact on them. To maintain their well‐being, which was essential for supporting their loved ones, CPs employed various approaches to alleviate their stress and find comfort.

#### 4.3.1 ‘A Forever Imprint on Me—I Have Never Felt So Useless’: Deep and Lasting CP Emotions and Physical Toll

CPs described the depth of the emotional and physical toll of being separated from the patient. CPs used words like *terrified, scared* and *alone* when reflecting on this time. Rebecca eloquently disclosed:

…the experience left a forever imprint on me…. I have never felt so useless and so helpless and that everything was beyond my control. The guilt was so huge.

Amanda understood the reason why she could not be with her husband, but still felt like she was failing him when he needed her the most. CPs of patients with progressive medical conditions described the profound stress that came from not knowing if they would see the patient in person again. This was the unfortunate reality for three CPs whose loved ones died alone during their admission. While any communication with the patient was longed‐for, CPs found it difficult to control their emotions when talking on the telephone with their loved ones. Brenda reflected on her feelings of helplessness when she called her husband every evening, knowing he could not respond due to a breathing tube. Rebecca revealed her ongoing need to unpack these complex feelings, and some CPs commented on the physical toll (i.e., exhaustion and immune conditions). Many CPs put their own needs second to those of the patient during this time; Teresa described how her total focus was on her husband during his admission, and her needs went neglected.

#### 4.3.2 ‘I Needed to Stay Close, In Case’: Resilience of CPs and Their Coping Strategies

Many CPs noted that it was essential to manage their own well‐being to effectively support their loved one during hospitalisation, despite the challenges of maintaining such a balance. CPs had strategies to maintain their wellness while adapting to the challenges posed by the Covid‐19 pandemic. Many relied on family support, seeking assistance from HCPs within their family to explain patient conditions and assist in navigating the healthcare system. In contrast, other CPs chose to distance themselves from well‐meaning family members who became a source of stress with constant phone calls requesting updates on the patient's condition. CPs with previous experience with the healthcare system were more at ease in this situation and framed it from a pragmatic perspective. Rose's husband had been in and out of the hospital multiple times before, and she felt this was just another visit. Many CPs found that keeping busy and continuing with their usual daily activities allowed them to focus on something other than their separation from the patient. Some admitted they found comfort in remaining as physically close to their admitted loved one as possible, even if they could not justify the logic of it. This might mean sitting in their car in the hospital parking lot, knowing they were close if something happened and the patient needed them. Amanda illustrated this in sharing:

I don't really know why I went and sat in my car [in the hospital parking lot] and hung out, but I just felt that … I needed to stay close by, just in case…

## 5 Discussion

Our study about the safety experiences of CPs when they were unable to be present with patients during their inpatient admission contributes to the understanding of CPs' perspectives on their safety engagement and the role that CPs may have in preventing patient harm. Overall, the findings suggest CPs value being physically present with patients during admissions, allowing them to see for themselves whether the patient is safe. Participants shared that their absence from the bedside led to disruptions in communication with the patient, and the HCP heightened their concerns for patient safety and negatively impacted their emotional and physical well‐being. To remain part of the patient's care, CPs had to rely on telephone communication, which, when ineffective, left CPs with doubts about the patient's safety. Not having regular updates about the patient's condition did not allow for a comprehensive understanding of the patient's condition, hindering the ability of the CP to advocate for the patient and their safety. Additionally, CPs feel they have a role in preventing AEs, sharing that they believe they could have prevented the AEs had they been present. The experience of being distanced from the patient was highly emotional, leading to ongoing stress and anxiety for the CP.

The 5‐Facet Framework described the important aspects of patient engagement in safety as identified by patients [19]. Notably, the relationship facet highlights the crucial role of CPs in preventing hospital harm, with patients relying on them as a *secondary safety check* (pg.1129) during their admission. Based on the results of this study, it appears that CPs are willing to accept this role in harm prevention, highlighting the importance of CPs' engagement as a safety strategy.

### 5.1 Communication Is Crucial

In our study, every CP described the challenges when trying to communicate from beyond the hospital with the patient and the HCPs. The disruptions in communication impacted the CPs' understanding of the patient's medical status, treatment plans and prognosis. This disruption negatively impacted CP's capacity to effectively advocate for the patient [47], a role emphasised in the 5‐Facet Framework as one that patients depend on CPs for during their admission [19]. This is consistent with other research findings indicating CPs want updates and information about the patient, favouring communication that is brief, regular and clear [7, 47]. Notably, despite the rapid increase in virtual ways to communicate with patients, CPs shared that the patient could not always use technology. This finding aligns with that of authors who have described shortcomings with the use of personal technology during Covid‐19, as HCPs often did not have the time to support patients, and there were inequalities with access to devices [48], and many patients were too unwell to communicate via technology [7, 24].

It has been found that regular communication between the CP and the patient decreased psychological distress, as the CP could verify the safety of the patient [7]. This was highlighted by several participants, who noted the inability to communicate with the patient caused significant stress, hindering the CP's ability to understand the patient's condition and treatment. In the absence of physical presence, CPs relied entirely on verbal communication and when lacking led to speculation and unfounded concerns about the patient's safety. This finding underscores the need for strong communication channels between patients and CPs, enabling CPs to stay engaged in patient care.

### 5.2 Increased Patient Safety Concerns When CPs Are Absent

The participants in our study expressed heightened concerns about patient safety, given that they were not present with the patient. CPs could not act as a second set of eyes and ears; they could not observe the care provided, monitor the patient for deterioration or advocate for the patient as needed. Consistent with previous studies, this led to worry and a lack of trust in the quality of care [49, 50]. If they choose to do so, CPs can be an important in‐person ‘layer of protection' as they can identify deficits in care and raise concerns to HCPs. Several CPs shared how their loved one experienced an AE during their admission, asserting the event—and resulting patient injury—could have been prevented or minimised had they been with the patient. Previous studies demonstrated that, although the overall incidence of AEs did not increase during the pandemic, there was a shift in the type of events that occurred; specifically, there was an increase in patient falls [51, 52], which are events that CPs have been found to effectively prevent [53].

### 5.3 CPs' Absence Impacted Their Physical and Emotional Well‐Being

Many of the CPs in our study reported that being separated from the patient during their time of need affected their physical and emotional well‐being. They described feelings of exhaustion and a worsening of pre‐existing conditions, which may have stemmed from their intense focus on the patient's well‐being at the expense of their own. These experiences illustrate the importance CPs place on being at the bedside with the patient during their admission. CPs feel they should be present with the patient to engage in their care and keep them safe, and when unable to do so, it leads to feelings of anxiety and stress. Recognising the importance of maintaining their own well‐being, several CPs shared the strategies they employed to ensure their health, enabling them to continue offering support to the patient. Many CPs garnered support from family members, rested and kept active, strategies that are supported in the literature [54].

Despite the growing number of studies on patient safety, the engagement of CPs in this area has garnered less attention, and they are often ‘blended in' with patients, without receiving dedicated focus. While there is recognition of the supportive role CPs can have in patient safety, additional research is essential to fully understand their contributions and identify the most effective ways to involve them. The findings of this study indicate that further exploration is needed regarding communication between the HCP and the CPs as a strategy to enhance safety, as well as methods to mitigate the stress often associated with the CP role. A greater understanding of how to effectively engage CPs in safety will allow for a more meaningful safety partnership, benefiting the CP and the patient.

### 5.4 Strengths and Limitations

This study has several strengths. It is an in‐depth qualitative study exploring CP experiences of safety during a period of visitor restrictions due to Covid‐19. Capturing the experiences of CPs during this time is important and a novel way to understand the unintended implications such restrictions have on patient safety and CPs. A strength of this study is the singular focus on CPs of adult patients. While research exists about CPs of paediatric patients, there is a limited understanding of the CP experience with adult patients. The size of our sample could be viewed as a limitation due to its size (12 participants); however, as no new substantive information was forthcoming, it was deemed appropriate to stop recruitment. Only two of the 12 participants identified as male, and all the CPs in this study described themselves as white. More varied experiences may have been shared with a more diverse sample; future research should endeavour to have a more diverse sample. Consequently, the findings may not be transferable to all CPs; however, they still provide critical perspectives on the unique challenges faced during this unprecedented time.

## 6 Conclusion

We sought to understand the safety experiences of CPs of admitted adult patients when visitor restrictions were imposed due to Covid‐19. Participants shared that their absence from the patient's bedside led to disruptions in communication, heightened their concerns for patient safety, and negatively impacted their emotional and physical well‐being. Additionally, many expressed feelings of helplessness regarding AEs affecting their loved ones, believing they could have intervened had they been present. This study affirmed the significant connectedness CPs have in ensuring patient safety and mitigating risk, enabling future work to better support their involvement. The safety of patients must remain paramount, with careful consideration of the risks of eliminating the opportunity for CPs to be at the bedside.

## Author Contributions

**Kayley Perfetto:** study conception, protocol development, data collection and analysis, manuscript drafting, manuscript review, approval for final submission. **Lenora Duhn:** study conception, protocol development, supervision, manuscript review, approval for final submission. **Kim Sears:** study conception, protocol development, manuscript review, approval for final submission. **Jane O'Hara:** conceived the study, developed the protocol, manuscript review, approval for final submission.

## Ethics Statement

This study was approved by the Queen's University Health Sciences and Affiliated Teaching Hospitals Ethics Board (HSREB#: 6040047).

## Consent

All participants provided written consent for the interview in which they participated to be recorded and confirmed they understood the interview would be transcribed verbatim with all information anonymised.

## Conflicts of Interest

The authors declare no conflicts of interest.

## Data Availability

To protect participant anonymity, transcripts will not be fully shared. Requests for data excerpts can be considered individually by contacting the corresponding author.
